# Mr. Charles Burnett

**Published:** 1910-06-18

**Authors:** 


### OBITUARY.

The death is announced of Mr. Charles Burnett, M.B., of Biggleswade, at the age of 72. Mr. Burnett was a member of the University of Aberdeen, where he gained the degree of M.S. in 1864, having also graduated M.B. At the time of his death he had retired from practice.

